# A Rare Case of a Hypervascular Placental Polyp Leading to Massive Postpartum Hemorrhage Requiring Hysterectomy

**DOI:** 10.1155/crog/4120029

**Published:** 2025-06-25

**Authors:** Samantha Kegel, Meena Dhir, Karina Hew, Chanda Reese, Gregory Lewis

**Affiliations:** ^1^Department of Obstetrics and Gynecology, University of Florida, Jacksonville, Jacksonville, Florida, USA; ^2^Mercer University School of Medicine, Savannah, Georgia, USA

**Keywords:** hysterectomy, placental polyp, postpartum hemorrhage

## Abstract

A placental polyp is a retained fragment of placental tissue that can lead to postpartum hemorrhage or become a nidus for infection. Hypervascular placental polyps can pose an increased risk of life-threatening postpartum hemorrhage requiring immediate intervention. Thus, prompt recognition and appropriate management are crucial in preventing maternal morbidity and mortality. Here, we present the case of a 29-year-old patient who had a spontaneous vaginal delivery at 36-week gestation after induction of labor due to pre-eclampsia with severe features. Quantitative blood loss at delivery was 1300 mL, and the patient received uterotonic medications. Due to continued bleeding, she underwent a suction curettage with clots and retained tissue removed from the uterine fundus. The total blood loss was estimated to be 4 L, and the massive transfusion protocol was activated. On postpartum Day 1, she underwent a bilateral uterine artery embolization; however, she developed further heavy vaginal bleeding. A second suction curettage was performed after ultrasound showed hypervascular material in the uterine cavity. The patient was subsequently discharged, but represented on postpartum Day 15 with increased bleeding. Imaging again demonstrated a hypervascular intrauterine polypoid mass. The patient desired definitive management and underwent a minimally invasive total hysterectomy.

## 1. Introduction

Placental polyps are remnants of normal placental tissue that is retained in the uterine cavity after delivery. They may lead to postpartum hemorrhage (PPH) or become a nidus for infection [1]. They are often discovered after a normal delivery or an incomplete abortion and can persist for months leading to abnormal uterine bleeding [2]. Hypervascular placental polyps are of clinical significance as they can lead to severe life-threatening hemorrhage and may require procedures such as uterine artery embolization, dilation and curettage, hysteroscopic resection, or hysterectomy [3]. A high index of suspicion with prompt recognition and management is vital in preventing dangerous PPH. To add to the body of literature of this very rare pathology, we highlight the management of a case of hypervascular placental polyp leading to PPH.

## 2. Case Presentation

**Figure 1:**
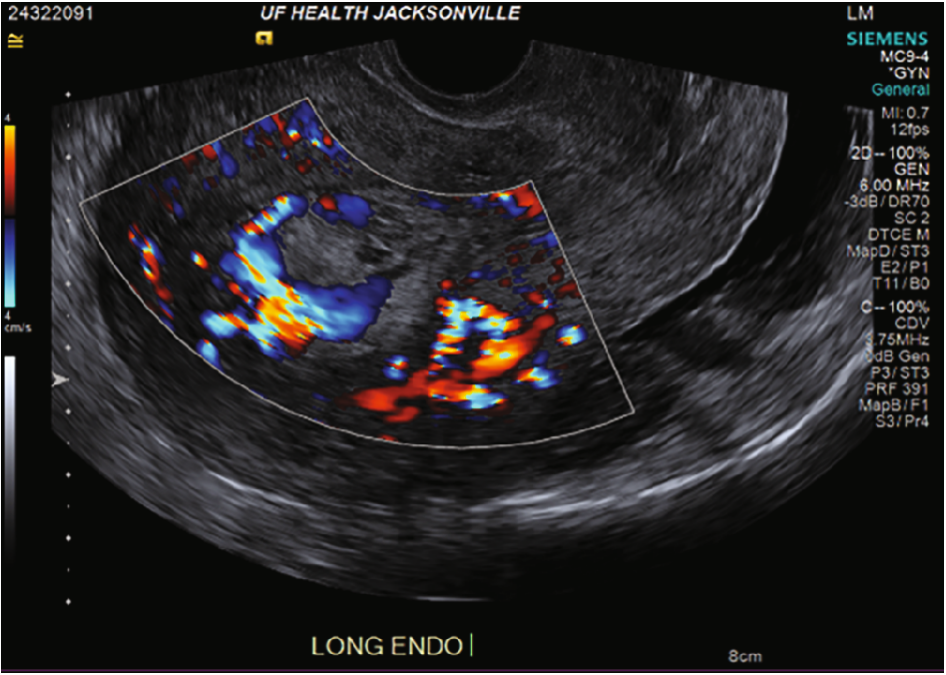
Transvaginal ultrasound demonstrating a thickened and heterogenous endometrium measuring 2.7 cm with hypervascularity.

**Figure 2:**
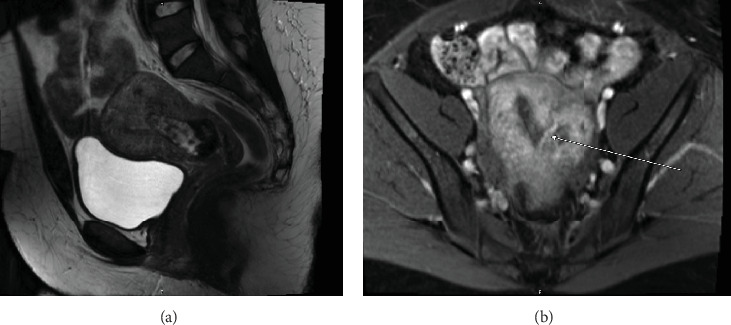
MRI pelvis with contrast. (a) Sagittal and (b) axial views demonstrating a 4-cm intrauterine mass with feeding vessels and areas of acute hemorrhage and hematoma.

**Figure 3:**
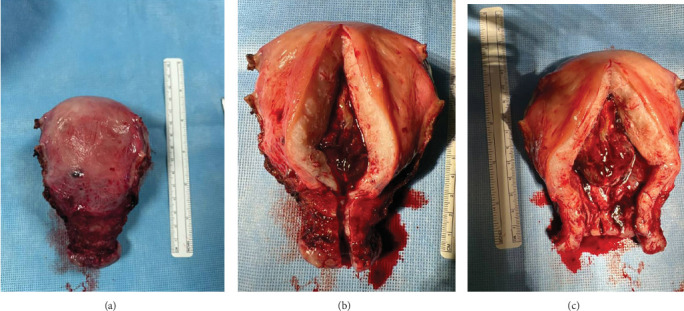
(a) Gross appearance of the uterus after hysterectomy. (b, c) Pedunculated intrauterine mass comprised of retained placental tissue with hemorrhage and calcifications consistent with a placental polyp. Unremarkable myometrium and unremarkable uterine serosa.

The index case is that of a 29-year-old G2P0010 patient who conceived via in vitro fertilization (IVF). She presented to the labor and delivery unit with elevated blood pressures at 36-week gestation. The patient ruled in for pre-eclampsia with severe features by blood pressure criteria. Her induction of labor was started with buccal misoprostol and a foley balloon and subsequently augmented with oxytocin. She then progressed to a spontaneous vaginal delivery of a live female infant. A significant PPH due to uterine atony occurred with 1300 mL of blood loss. The PPH was managed with several uterotonics including intravenous oxytocin, rectal misoprostol, and four doses of carboprost, as well as tranexamic acid. Due to further vaginal bleeding, a suction curettage was performed with the removal of placental tissue. The curettage was performed under ultrasound guidance, and at the conclusion of the case, the endometrial stripe was noted to be thin and free of retained products of conception. The total blood loss at the time was 4 L and required massive transfusion protocol. On postpartum Day 1, the patient again developed heavy vaginal bleeding, and a bilateral uterine artery embolization was performed. On postpartum Day 5, she experienced further episodes of heavy vaginal bleeding and underwent a second suction curettage after pelvic ultrasound demonstrated vascularized tissue in the uterine cavity. After the procedure, the patient's bleeding subsided, and she was discharged in stable condition. Serum bHCG was declining. The patient represented on postpartum Day 15 with persistent heavy vaginal bleeding. A transvaginal pelvic ultrasound showed a hypervascular endometrial mass (Figure 1); this was further characterized by a pelvic MRI to be a 4-cm polypoid appearing mass with areas of hemorrhage (Figure 2). After discussing the MRI results with the patient, the decision was made to proceed with hysteroscopic evaluation of the lesion. If the lesion was unable to be resected hysteroscopically, the patient desired to proceed with hysterectomy, as she did not wish to contend with further bleeding episodes. At hysteroscopy, we identified a hypervascular polypoid mass on the posterior uterine wall. Due to the hypervascular nature of the mass and brisk hemorrhage on contact, the decision was made to move forward with a total laparoscopic hysterectomy and bilateral salpingectomy. The procedure was uncomplicated, and the patient was discharged in stable condition on postoperative Day 1. At a 6-week postpartum visit, the patient was clinically stable and recovering well. The final pathology reported a pedunculated uterine mass with retained placental tissue, hemorrhage, and calcifications (Figures 3, 3, and 3).

## 3. Discussion

Hypervascular placental polypoid masses (HPPMs) are remnants of placental tissue that undergo neovascularization and create a nidus for infection and PPH. HPPM is identified in 0.25% of pregnancies and causes life-threatening PPH in 6% of identified cases [1]. They most commonly occur after spontaneous vaginal delivery or elective pregnancy termination. HPPM can be classified into two types: acute (less than 4 weeks postpartum) and chronic (more than 4 weeks postpartum) [4]. The acute type is far more common [5]. Although management guidelines do not currently exist, prompt recognition and treatment are crucial to prevent PPH.

Pathogenesis of HPPM often differs based on the antecedent pregnancy outcome. HPPM following spontaneous deliveries favors fibrinosis, while those following elective abortions lead to preservation of the synctiotrophoblastic layer of villi. These syncytiotrophoblasts promote neovascularization and have anticoagulative properties. In addition, hyalinization of neovessels prevents contraction, further increasing the risk of uterine atony and subsequent hemorrhage, as was seen in our case [6]. Predisposing factors for development of HPPM include prior disruption of Nitabuch's layer (including, but not limited to, prior uterine instrumentation, Asherman's syndrome, and placenta accrete spectrum), creating a surface for firm adherence to the myometrium and subsequent neovascularization [6].

It is important to identify patients at increased risk of HPPM at the time of hospital admission and include HPPM in the differential diagnosis in the event of a PPH. The ability to differentiate HPPM from other causes of PPH, including retained placenta and arteriovenous malformations (AVMs), is crucial to ensure appropriate treatment. While AVM and retained placenta are often static lesions, HPPM develops over time as it neovascularizes [4]. In addition, AVM forms within the myometrium rather than the endometrial canal [7]. HPPM can be further differentiated from retained placenta due to the presence of feeding vessels.

In our case, intraoperative ultrasound after the first curettage demonstrated an empty uterine cavity. The lesion developed later, as repeat imaging on PPD 5 and 15 revealed a hypervascular endometrial polyp, supporting the diagnosis of HPPM.

Beyond clinical suspicion, advanced imaging is often useful in diagnosis of HPPM. Pelvic ultrasonography with power Doppler is helpful for the initial diagnosis [3]. In addition to identifying hypervascular intrauterine contents, studies have demonstrated that power Doppler ultrasonography may be helpful in risk stratification of HPPMs based on degree of hypervascularity [2]. CT angiography and MRI are also useful in evaluation of hypervascularity and myometrial involvement, as well as follow-up after treatment [8, 9].

Although definitive management guidelines for HPPM have not yet been developed, sharp uterine curettage and hysteroscopy are commonly used for first-line management. Hysteroscopy allows for direct visualization of the HPPM for more precise excision and is becoming more favorable in practice [10]. In a patient with uncontrollable hemorrhage, which may preclude intracavitary visualization, uterine artery embolization or temporary iliac artery occlusion can be considered [3]. However, in the setting of intractable hemorrhage unresponsive to all other interventions, hysterectomy may be necessary, as was seen in our case. While cases of HPPM requiring hysterectomy have been previously demonstrated in the literature, our case highlights obstacles in prompt identification and treatment of HPPM as well as the persistent and elusive nature of this pathology. Specifically, the patient underwent two suction curettages, a uterine artery embolization and a hysteroscopy prior to definitive management of HPPM.

In summary, HPPMs are rare sequelae of spontaneous deliveries and abortions, characterized by trophoblastic proliferation and neovascularization, that can result in PPH. Prompt recognition and management are crucial in preventing morbidity and mortality related to HPPM. Here, we present the case of HPPM leading to prolonged PPH ultimately necessitating hysterectomy.

## Data Availability

Data sharing is not applicable to this article as no new data were created or analyzed in this study.

## References

[1] Alhussami R., Noorwali F., Ibrahim G. (2020). A Rare Medical Dilemma: Presentation and Management of Placental Polyp. *Cureus*.

[2] Mori M., Iwase A., Osuka S. (2016). Choosing the Optimal Therapeutic Strategy for Placental Polyps Using Power Doppler Color Scoring: Transarterial Embolization Followed by Hysteroscopic Resection or Expectant Management?. *Taiwanese Journal of Obstetrics and Gynecology*.

[3] Marques K., Looney C., Hayslip C., Gavrilova-Jordan L. (2011). Modern Management of Hypervascular Placental Polypoid Mass Following Spontaneous Abortion: A Case Report and Literature Review. *American Journal of Obstetrics and Gynecology*.

[4] Spielvogel R. (2023). A Hypervascular Placental Polyp After Complete Abortion: A Case Report. *BMC Womens Health*.

[5] Watcharotone W., Leelaphatanadit C. (2005). Placental Polyp: A Case Report. *Siriraj Medical Journal*.

[6] Milovanov A. P., Kirsanov Ia N. (2008). The Pathogenesis of Uterine Hemorrhages in the So-Called Placental Polyps. *Arkhiv Patologii*.

[7] Ishihara T., Kanasaki H., Oride A., Hara T., Kyo S. (2016). Differential Diagnosis and Management of Placental Polyp and Uterine Arteriovenous Malformation: Case Reports and Review of the Literature. *Women's Health*.

[8] Umezu T., Iwase A., Ota T. (2010). Three-Dimensional CT Angiography Is Useful for Diagnosis of Postabortion Uterine Hemorrhage: 3 Case Reports and Review of the Literature. *Journal of Minimally Invasive Gynecology*.

[9] Kurachi H., Maeda T., Murakami T. (1995). MRI of Placental Polyps. *Journal of Computer Assisted Tomography*.

[10] Takeda A., Koyama K., Imoto S., Mori M., Sakai K., Nakamura H. (2010). Placental Polyp With Prominent Neovascularization. *Fertility and Sterility*.

